# A Synchronization-Driven Learning Rule for Pattern Separation in Self-Organizing Probabilistic Spiking Neural Networks

**DOI:** 10.21203/rs.3.rs-9917769/v1

**Published:** 2026-07-14

**Authors:** Faramarz Faghihi, Ahmed Moustafa, Samuel Neymotin

**Affiliations:** 1 Department of Medical Physiology, Division of Heart & Lungs, University Medical Center Utrecht, Utrecht, The Netherlands; 2 School of Psychology, Faculty of Society and Design, Bond University, Gold Coast, Queensland, Australia; 3 Department of Human Anatomy and Physiology, the Faculty of Health Sciences, University of Johannesburg, South Africa; 4 Center for Biomedical Imaging & Neuromodulation, Nathan Kline Institute for Psychiatric Research, Orangeburg, NY; 5 Department of Psychiatry, NYU School of Medicine NY, NY

**Keywords:** Pattern Separation, Probabilistic Spiking Neural Networks, Synchronization-Based Learning, Non-Hebbian Plasticity, Excitation–Inhibition Balance, Cognitive Robotics

## Abstract

Neuroscience-inspired neural networks provide a promising framework for bridging biological principles and adaptive artificial intelligence systems. Here, we propose a novel synchronization-based synaptic learning rule for self-organizing probabilistic spiking neural networks (PSNNs) with feedback inhibition. In the proposed model, synaptic plasticity is regulated by the temporal synchronization of presynaptic spike activity of single neurons, enabling unsupervised adaptation of synaptic weights and network connectivity. We systematically investigated how feedback inhibition influences network dynamics, stability, synchronization, and pattern separation efficacy. The results revealed that moderate inhibition produces an optimal balance between excitatory and inhibitory activity, maximizing pattern separation while preventing both excessive excitation and over-suppression of network activity. Comparative analysis further demonstrated that the proposed synchronization-based learning mechanism outperforms conventional Hebbian learning in achieving efficient and stable pattern separation in this neural network. Finally, the trained network was embedded in a simulated autonomous agent navigating a two-dimensional environment, where it successfully identified and avoided a learned obstacle pattern. These findings highlight the critical role of inhibitory regulation and synchronization-driven plasticity in self-organizing spiking systems and support the potential application of biologically inspired learning mechanisms in computational neuroscience, neuromorphic computing, and cognitive robotics.

## Introduction

Neuroscience-inspired artificial intelligence (AI) draws on the structure and function of the brain to develop adaptive and efficient computational models. By emulating mechanisms such as spiking activity, synaptic plasticity, and network organization, this approach has led to advances including Spiking Neural Networks (SNNs), neuromorphic hardware, and brain-inspired learning algorithms, enabling energy-efficient and biologically plausible computation^1^. SNNs capture neural dynamics with high temporal precision and provide a framework for studying learning and plasticity^2,3^. Their event-driven nature also makes them well suited for neuromorphic systems, supporting applications in robotics such as navigation, object recognition, and motor control^4,5^.

Probabilistic Spiking Neural Networks (PSNNs) integrate the principles of probabilistic computation with the dynamics of spiking neurons. Instead of producing deterministic outputs given an input, these networks model uncertainty by incorporating randomness in neural responses such as spike timing, firing probability, or synaptic transmission^6,7^. This stochastic behavior enables PSNNs to represent and manipulate uncertainty just as biological neural systems do in tasks like perception, prediction, and decision-making^8,9^. PSNNs offer a computationally efficient alternative to traditional integrate-and-fire (I&F) models, particularly in large-scale or inference-driven applications. Unlike I&F neurons, which require continuous tracking of membrane potentials through numerical integration, probabilistic models generate spikes based on simplified statistical rules, often driven by input rates or instantaneous likelihoods. While I&F models provide greater biological realism and temporal precision, PSNNs are increasingly used in neuromorphic computing and probabilistic inference tasks where efficiency and scalability are key priorities^10,11,12^.

The balance between excitatory (E) and inhibitory (I) neuronal activity is fundamental to normal brain function^13,14^. This balance ensures that neural circuits operate within an optimal range, preventing excessive excitation that can lead to hyperactivity or seizures, and avoiding excessive inhibition that can suppress necessary neural signaling^15,16^. Disruptions to the balance and synchronization between inhibitory and excitatory neuronal populations can arise from altered synaptic strengths, dysregulation of neurotransmitters, and neurodevelopmental abnormalities affecting inhibitory interneuron development^17,18^. E/I imbalances impair the fine-tuned neural dynamics required for efficient information processing^19,20^. Studies have shown that disrupted E/I balance, particularly during resting-state brain activity, contributes to memory loss and cognitive decline in patients with Alzheimer’s disease^21,22^. Therefore, understanding these mechanisms provides important insights for therapeutic strategies aiming to restore E/I balance and improve cognitive function in neurological and psychiatric disorders.

Pattern separation is a neural computation that enables the brain to distinguish between similar inputs or experiences by creating distinct representations^23,24,25^. This function, primarily associated with the hippocampus and the dentate gyrus, is essential for episodic memory, spatial navigation, and learning^26,27,28^. It ensures that overlapping stimuli or events do not interfere with one another, thus preventing memory confusion and supporting accurate recall. Computational studies and simulations have been developed to investigate parameters that are not easily accessible through experimental approaches^29,30,31^. These parameters include neuronal biophysical properties, synaptic learning rules, neuronal population size, and network connectivity^32,33,34^.

In cognitive disorders such as Alzheimer’s disease and schizophrenia, impaired pattern separation is linked to dysfunction in hippocampal circuits, particularly the dentate gyrus. This disruption leads to overlapping neural representations and difficulty distinguishing similar memories or contexts. In Alzheimer’s disease, neurodegeneration and synaptic loss impair the formation of distinct memory traces, while in schizophrenia, altered excitatory–inhibitory balance reduces encoding fidelity. Together, these deficits result in memory interference, reduced learning precision, and impaired adaptive behavior^35,36^.

The balance between excitatory (E) and inhibitory (I) neuronal activity is crucial for efficient pattern separation in biological neural systems. In regions such as the hippocampus, particularly the dentate gyrus, lateral inhibition and well-tuned E/I ratios help to separate similar input patterns, reducing overlap and enhancing discrimination. When excitation and inhibition are out of balance, either through weakened inhibition or excessive excitation, the capacity for separating similar inputs deteriorates, leading to less reliable memory representations and impaired cognitive performance^30,37^.

In artificial neural networks, non-Hebbian learning rules refer to mechanisms of synaptic weight adjustment that do not follow the classical Hebbian principle of ‘cells that fire together wire together’. Instead, these rules rely on alternative factors such as global error signals, activity from surrounding neurons, or temporal constraints^38,39^. A prominent example is the backpropagation algorithm, which updates weights based on the gradient of a loss function with respect to network output^40,41^. Unlike classic Hebbian rules that rely on the precise co-activation of pre- and postsynaptic neurons, these mechanisms are governed by global feedback signals or intrinsic activity constraints, enabling broader regulation of synaptic efficacy^42,43^. These rules allow artificial systems to learn more flexibly and robustly than would be possible with Hebbian mechanisms alone, supporting applications such as pattern recognition, motor control, and adaptive behavior in dynamic environments^44^.

Synchronization-based information transfer refers to the process by which neurons receive and interpret information not just from the rate of incoming spikes, but from their precise timing and temporal coordination^45^. When multiple presynaptic neurons fire in a synchronized manner, their combined inputs can summate more effectively at the postsynaptic neuron, making it more likely to generate a spike. This temporal alignment enhances the signal-to-noise ratio and allows single neurons to act as coincidence detectors, responding selectively to coordinated patterns of input^46^.

Neuronal synchronization plays a critical role in various neural computations, including sensory processing, attentional modulation, and temporal coding. For example, in the visual and auditory systems, the synchronization of input spikes can help encode stimulus features with high temporal precision. In cortical circuits, gamma-band synchronization (30–80 Hz) is thought to dynamically route information across neural populations, allowing individual neurons to selectively respond to inputs from specific, coherently active groups^47,48^.

During the early stages of neurodevelopment, the formation of synaptic connections is largely guided by activity-independent processes such as molecular signaling, structural plasticity, and spontaneous neural activity, rather than by activity-dependent mechanisms like Hebbian learning. At this stage, Hebbian learning may be less effective because sparse and immature connectivity does not yet provide sufficient coincident activity to drive synaptic strengthening. Instead, mechanisms based on synchronization and structural remodeling are likely to play a dominant role in establishing the initial network architecture^49^. Only after a sufficient level of connectivity has been achieved does Hebbian learning become more effective in refining and stabilizing synaptic connections^50^.

Motivated by this biological perspective, we propose a synchronization-based learning rule that captures key aspects of early neurodevelopment, particularly the formation of neural circuits in the developing brain. This rule is implemented in a probabilistic four-layer spiking neural network with two inhibitory layers providing feedback. Guided by biologically inspired wiring principles, the model self-organizes its inter-layer connectivity in response to input patterns during training, enabling autonomous and unsupervised adaptation.

We describe the architecture of the neural network and its underlying probabilistic mechanisms, followed by a series of unsupervised learning experiments focused on basic pattern separation. These experiments investigate the influence of various parameter values, particularly the intensity of inhibition. Finally, the optimized neural network is integrated into a simulated robot tasked with navigating a two-dimensional environment while avoiding previously learned stimuli.

## Methods

### Network architecture

**Fig. 1 |:**
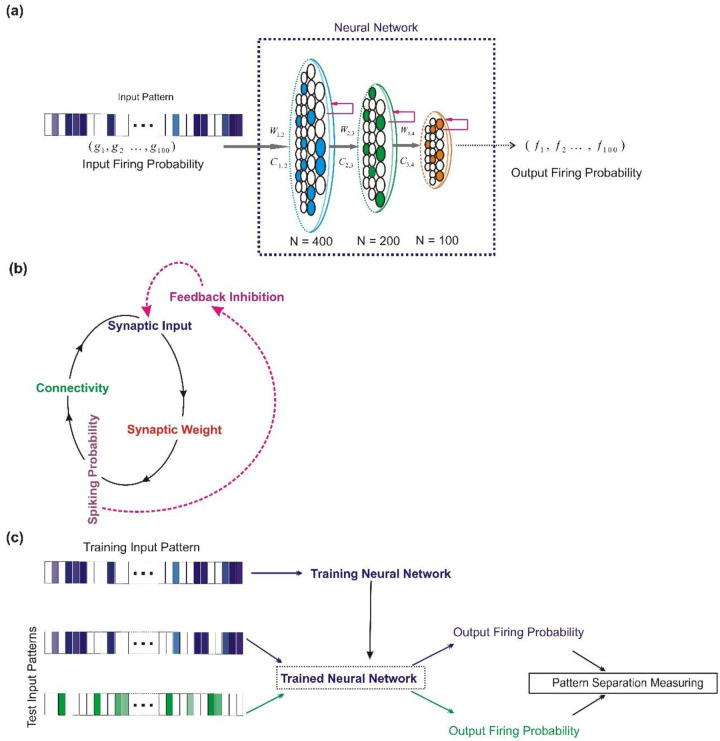
Model architecture, network dynamics, and simulation paradigm. (**a**) Architecture of the proposed Probabilistic Spiking Neural Network (PSNN). The model consists of a stimulus layer followed by three excitatory probabilistic spiking layers and three corresponding inhibitory layers. Each excitatory neuron is paired with a dedicated inhibitory neuron that provides feedback inhibition. Synaptic weights and connection probabilities are initially set to 0.05 and are updated dynamically during training. The firing rates of neurons in the fourth (output) excitatory layer are used to evaluate pattern separation performance. (**b**) Schematic illustration of network dynamics during training. Synaptic inputs influence neuronal spiking activity, which in turn drives changes in synaptic weights through the synchronization-based learning rule. The spiking probability of each neuron depends on its total excitatory input, determined by presynaptic activity, synaptic weights, and network connectivity. Connectivity is updated as a function of postsynaptic spiking probability, forming a closed-loop interaction between activity, connectivity, and synaptic plasticity. Feedback inhibition modulates synaptic input according to the inhibition parameter (μ), thereby regulating network stability. (**c**) Simulation paradigm for evaluating pattern separation. The network is first trained using a predefined input pattern (blue). Following training, the learned network is presented with both the training pattern and novel test patterns. Output firing-rate vectors are computed for each pattern, and the normalized distance between these vectors is used as a quantitative measure of pattern separation efficacy under different parameter settings.

Inspired by cortical architecture and hippocampal circuits, we developed a four-layer feed-forward Probabilistic Spiking Neural Network (PSNN) composed of both excitatory and inhibitory neurons (Fig. 1a). The first layer consists of 100 sensory neurons that encode input stimuli.

In the experimental setup, stimulus pattern are defined as fixed subsets of neuron indices, each representing a distinct input condition. During training, stimulus patterns are presented during each epoch over 100 time bins. Within each stimulus pattern, only the corresponding neurons are active, whereas the remaining neurons remain quiescent. Spike generation for each active neuron follows a Bernoulli process with a firing probability p ∈ [0,1], independently sampled for each neuron at the beginning of the simulation and held constant throughout the simulation. Thus, an active neuron emits a spike (denoted as 1) with probability ‘p’, and remains silent (as 0) otherwise. For the pattern separation experiments, all stimulus patterns are constructed to be fully disjoint, i.e., with no overlapping active neurons between any pair of patterns. This ensures orthogonality in the input space, enabling a rigorous assessment of the network’s capacity to form distinct internal representations for non-overlapping inputs.

The second, third, and fourth layers consist of probabilistic spiking neurons whose activity is governed by the model dynamics. These layers comprise 400, 200, and 100 neurons, respectively. Each layer includes inhibitory neurons, with every excitatory neuron paired one-to-one with a corresponding inhibitory neuron. Inhibitory neurons do not interact with one another; instead, each inhibitory neuron connects exclusively to its paired excitatory neuron, providing probabilistic feedback inhibition via synaptic connections.

The synaptic weight between each excitatory neuron and its paired inhibitory neuron was fixed at 1 throughout all training and simulation phases.

At the beginning of network training, the probability of forming a connection between neurons in adjacent layers was set to 0.05. Similarly, initial synaptic weights were set to 0.05. Both connectivity and weights were dynamically adjusted during training.

### Synaptic learning rule

In this work, we introduce a novel synaptic learning rule inspired by early stages of neural development, when neurons receive only a few random synaptic inputs that are insufficient to independently trigger spikes. We hypothesize that synaptic weights are strengthened according to the degree of synchronization among presynaptic inputs. In this framework, synchronized synaptic activity directed toward a postsynaptic neuron is assumed to convey meaningful information about the external environment.

Synchronized inputs can encode critical environmental features because their timing strongly influences postsynaptic responses. When multiple presynaptic neurons release neurotransmitters simultaneously, or within a narrow temporal window, the postsynaptic neuron is more likely to fire, thereby providing a reliable mechanism for transmitting stimulus-related information. Such temporal synchronization enables the network to represent more complex signals and supports higher-level processes such as sensory perception, attention, and memory formation. Moreover, neuronal synchrony contributes to oscillatory brain activity, which has been implicated in large-scale functions including motor control, cognition, and decision-making.

To formalize this non-Hebbian learning rule, we first compute the degree of synchronization among synapses converging onto a single postsynaptic neuron. Consider n presynaptic neurons, each connected by one synapse. Neurons fire stochastically according to their firing probabilities, producing spike trains represented as binary vectors, where “1” denotes a spike and “0” denotes silence. Spiking activity is evaluated over a 100-bin time window (one trial), after which synaptic weights are updated. The procedure is as follows:

For each time bin, we compute the mean spike activity of all presynaptic neurons (equation 1).

These mean values are transformed into a synchronization score for each time bin (equation 2). At each time bin, the presynaptic spike vector is multiplied by the corresponding synchronization score (equation 3).

Finally, the mean of these weighted values is calculated for each presynaptic neuron and used to update its synaptic weight at the end of the trial T (equation 4).

(1) $$Ms_{t}=\frac{\sum_{i}^{N}s^{t}}{N};$$

*where* N: number of neurons in the layer

(2) $${\text{syn}}_{t}=\frac{1}{1+\text{exp}\left(-\vartheta *Ms_{t}-\varepsilon \right)};\;\text{where}\;\varepsilon =3;\;\vartheta =0.5$$

(3) $${\text{score}}^{j}=\frac{\sum_{t=1}^{L}\left({\text{syn}}_{t}*s_{j}^{t}\right)}{L};\;\text{where}\;S_{j}^{t}:\;\text{spike}\;\text{of}\;\text{neuron}\;j^{\text{th}}\;\text{at}\;\text{timebin}\;‘\text{t}’\;\text{L}=100\text{;}$$

(4) $$w_{T+1}^{j}=w_{T}^{j}+\alpha *{\text{score}}^{j};\;\text{where}\;\alpha =0.003:\;\text{learning}\;\text{rate},\;\text{T}:\text{trial}\;\text{number}$$

### Neuron model and dynamics

The neuron model used in the proposed feedforward neural network is defined as follows. Neurons in the stimulus layer are predefined probabilistic units, connected to the second layer with an initial connection probability of 0.05. Neurons in the second, third and fourth layers are modeled as probabilistic spiking neurons, with their inputs defined by Equation 5.


(5) $$I_{j}^{t}=\sum_{i}^{n}\left(S_{i}^{t}*W_{ij}*C_{ij}\right)$$

n: number of connected synapses to the single neurons

$I_{j}^{t}$: total input into neuron $j^{\text{th}}$ at timebin ‘t’

$W_{ij}$: synaptic weight

$C_{ij}$: connectivity rate

The probability of spiking for each neuron at a given time bin is determined by Equation 6. The connectivity between neurons and the preceding layer is updated at the end of each training trial according to equations 8–10. Here, $M_{p}$ denotes the average spiking probability of a neuron at the end of a trial. Equation 9 is inspired by previous work that introduced a probabilistic model of structural plasticity, incorporating the biological relationship between synaptic weight and volume, as well as the dependency of volume on synaptic lifetime^51^.


(6) $$P_{j}^{t}=\frac{1}{1+\text{exp}\left(-\gamma \left(l_{j}^{t}-\delta \right)\right)}-H_{j}^{t}\;;\;\text{where}\;\delta =1;\;\gamma =3$$

(7) $$H_{j}^{t}=\text{exp}\left(-\frac{\left(1-\mu \right)}{P_{j}^{t}}\right)\;;\;\mu \in [0,1)\;;$$

(8) $$Mp=\frac{\sum_{t=1}^{L}P_{j}^{t}}{L};\;\text{L}=100,$$

(9) $$M=1-\text{exp}\left(-a^{2}*Mp^{\frac{4}{3}}\right);\;\text{where}\;\text{a}\in \left[0.1\;10\right]\;;$$

(10) $$C\left(T+1\right)=C\left(T\right)+\beta *M;\;\text{where}\;\beta =0.003\;\text{T}:\;\text{trial}\;\text{number}$$

In this framework, M represents the probability of forming new synapses and is used to calculate the updated connectivity rate of a single neuron to the preceding layer. As training progresses, modifications in neuronal connectivity alter the total input received by each neuron, which in turn affects its spiking probability, forming a closed-loop interaction between connectivity, neuronal activity, and synaptic plasticity (Fig. 1b).

To stabilize neuronal activity during training, feedback inhibition was implemented. Inhibitory neuronal activity is modeled by equation 7, where $H_{j}^{t}$ represents the spiking probability of an inhibitory neuron receiving input from a corresponding excitatory neuron at time bin t. The parameter μ controls the strength of inhibition (Fig. 5a). This inhibition probability is then used to modulate the input activity of the corresponding excitatory neuron.

Thus, both the connectivity and spiking probability of each neuron are iteratively updated across trials: connectivity is modified at the end of each trial, while feedback inhibition dynamically regulates excitatory inputs during training.

To evaluate pattern separation efficacy, we adopted the method described in a study on modeling pattern separation in dentate gyrus^30^. Specifically, fully separated training input patterns were presented to two independent models. Following training, the evolved connectivity and synaptic weights of excitatory neurons were used to calculate the ordered pattern of average firing rates in the third layer. Pattern separation efficacy under different parameter values was quantified by computing the normalized metric distance between these output patterns. Synchronization within the output layer excitatory and inhibitory neuronal populations was quantified using a Population Synchrony Index (PSI), based on temporal fluctuations in the population firing rate. Spike trains were represented as a binary matrix, where each entry indicates the presence or absence of a spike in a given time bin.

### Pattern separation measurement

The activity of the fourth (output) layer was used to quantify the pattern separation performance of the trained network (Fig. 1c). The network was trained using predefined parameter values and a given input stimulus pattern. Following training, the normalized distance between firing-rate vectors in the fourth layer was computed for the training pattern and for fully separated test patterns. The mean of these distances was used as a quantitative measure of pattern separation efficacy.

### Statistical analysis

All statistical analyses were performed using MATLAB R2022 (The MathWorks, USA). Quantitative results were obtained from n independent simulation trials initialized with different random seeds. Data are presented as mean ± standard error of the mean (S.E.M.). To evaluate the effect of inhibition intensity (μ) on pattern separation efficacy and firing rates, a one-way analysis of variance (ANOVA) was performed across inhibition conditions. When ANOVA revealed significant differences (p < 0.05), post-hoc multiple comparisons were conducted using Tukey’s honestly significant difference (HSD) test. Statistical significance was defined as p < 0.05. Significant differences are indicated in the figures by asterisks (*p < 0.05, **p < 0.01). The number of independent simulation trials (n) for each condition is reported in the corresponding figure captions.

### Experiments and Simulations

The developed model was evaluated for its pattern separation efficacy under varying levels of inhibitory intensity. We investigated the dynamics of key model parameters, including connectivity and synaptic weights, with particular attention to conditions of network stability and their relationship to separation performance. Furthermore, we examined synchronization within excitatory and inhibitory populations and its contribution to both network stability and pattern separation. These analyses provide important insights into the role of inhibition in cognitive disorders in which pattern separation is impaired, such as autism and schizophrenia.

The proposed PSNN architecture offers a biologically plausible framework for integrating perceptual learning and action selection in autonomous systems. Its event-driven, low-power characteristics make it well-suited for deployment in neuromorphic hardware, with potential applications in cognitive robotics and embodied artificial intelligence. Moreover, the model’s modular and self-organizing nature enables further extensions for complex cognitive tasks such as goal-directed navigation, adaptive behavioral switching, and interaction with dynamic environments.

In addition, the trained Probabilistic Spiking Neural Network (PSNN) was embedded within an artificial agent navigating a two-dimensional simulated environment, employing a random-walk exploration paradigm. The agent’s task was defined as obstacle avoidance, whereby a specific stimulus pattern learned during training was designated as the obstacle to be recognized and avoided. This stimulus was characterized by a unique, non-overlapping activation pattern of neurons, distinguishing it from other benign stimuli with entirely different neural representations.

During navigation, when the agent encountered a stimulus (i.e., came within a predefined spatial proximity to it), the PSNN processed the input and generated an internal representation based on previously learned patterns. If the stimulus matched the learned obstacle pattern, the agent inhibited forward motion and selected an alternative direction, thereby demonstrating recognition and context-dependent decision-making. In contrast, the agent was allowed to traverse areas associated with non-obstacle stimuli, as these elicited distinct neural activity patterns.

## Results

To investigate the dynamics of the proposed model and identify the conditions that optimize its pattern separation performance, we systematically varied key parameters, with particular emphasis on the strength of feedback inhibition. All experiments and simulations were performed using MATLAB (MathWorks Inc.), which provided the framework for neural network modeling, data analysis, and visualization. Across all simulations, feedback inhibition played a pivotal role in maintaining network stability by limiting excessive synaptic growth and connectivity during training. It also significantly contributed to enhancing the network’s ability to separate input patterns.

**Fig. 2.**
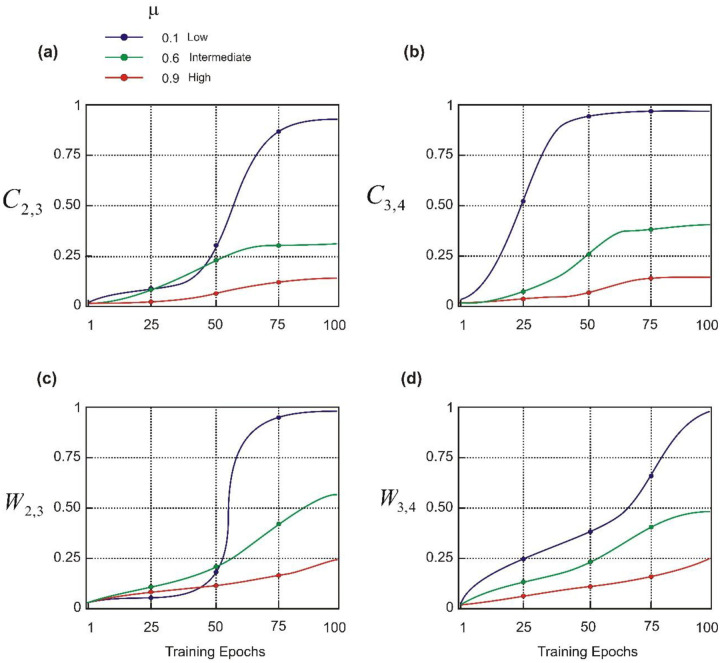
Dynamics of connectivity and synaptic weights during training under different levels of feedback inhibition. Mean connectivity rates between the second and third layers (**a**) and between the third and fourth layers (**b**), together with mean synaptic weights for the corresponding connections (**c, d**), are shown for three inhibition strengths: low (μ=0.1), moderate (μ=0.6), and high (μ=0.9). Feedback inhibition strongly influences the evolution of both connectivity and synaptic plasticity. Low inhibition promotes rapid growth and high saturation levels of connectivity and synaptic weights, whereas high inhibition markedly suppresses their development. Moderate inhibition produces balanced dynamics characterized by gradual growth and stable convergence, supporting controlled network self-organization during training.

We found that moderate levels of inhibition provided the most balanced regulation of network growth. Specifically, this regime effectively controlled both inter-layer connectivity and synaptic plasticity. In contrast, low inhibition led to excessive synaptic strengthening and highly saturated connectivity, whereas high inhibition strongly suppressed the growth of connectivity and synaptic weights, thereby limiting learning. Networks trained under moderate inhibition exhibited gradual and stable increases in connectivity rates between the second and third layers and between the third and fourth layers (Fig. 2a, b), as well as controlled growth of synaptic weights in the corresponding connections (Fig. 2c, d).

**Fig. 3.**
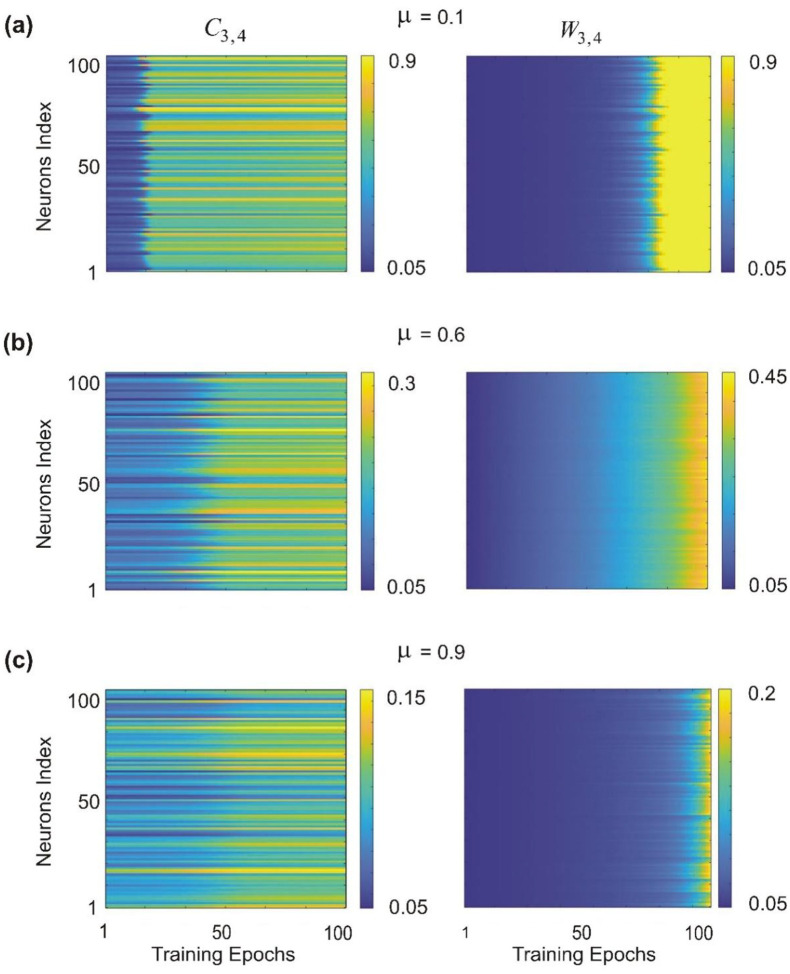
Evolution of connectivity C3,4 (left column) and synaptic weights W3,4 (right column) of fourth-layer neurons across training epochs under different inhibition strengths: (**a**) low inhibition (μ=0.1), (**b**) moderate inhibition (μ=0.6), and (**c**) high inhibition (μ=0.9). Rows correspond to neurons and columns to training epochs. Low inhibition leads to rapid growth and saturation of connectivity and synaptic weights, whereas high inhibition suppresses synaptic adaptation. Moderate inhibition promotes balanced and stable learning dynamics.

Figure 3 illustrates the evolution of connectivity and synaptic weights for neurons in the fourth layer under three levels of inhibitory intensity; similar trends were observed in the second and third layers. As training progressed, both connectivity and synaptic weights adapted independently for each neuron according to the presented input patterns (Fig. 3a–c). The inhibition level strongly influenced the learning dynamics. Under low inhibition (μ=0.1), connectivity and synaptic weights rapidly increased and approached saturation. In contrast, high inhibition (μ=0.9) substantially suppressed synaptic growth, resulting in weak connectivity and limited weight adaptation. Moderate inhibition (μ=0.6) produced the most balanced learning dynamics, supporting gradual synaptic development while avoiding both excessive saturation and insufficient growth.

**Fig. 4.**
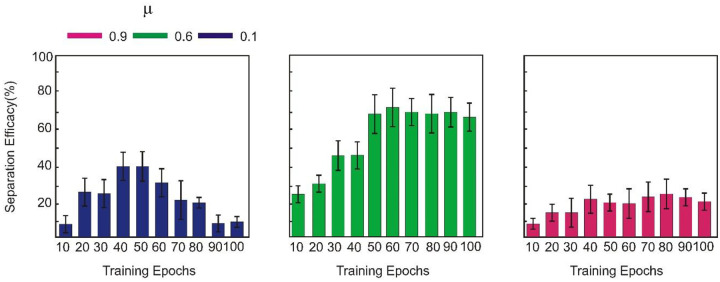
Pattern separation efficacy as a function of training epochs for three inhibition strengths: low (μ=0.1), moderate (μ=0.6), and high (μ=0.9). Error bars represent the standard deviation across trials. Pattern separation efficacy increases with training under moderate inhibition and reaches its highest values around epochs 60–70. In contrast, low and high inhibition levels result in substantially lower separation performance throughout training, indicating that a moderate level of inhibition provides the most favorable conditions for pattern separation.

Figure 4 shows that pattern separation efficacy depends strongly on the level of inhibition. The highest performance was obtained for moderate inhibition (μ=0.6), reaching approximately 70% around epochs 60–70. This improvement coincides with the balanced synaptic and connectivity dynamics observed in Figure 3. Under low inhibition (μ=0.1), connectivity and synaptic weights tended toward saturation, reducing the network’s ability to generate distinct representations and resulting in lower separation efficacy. Conversely, high inhibition (μ=0.9) excessively constrained synaptic growth, limiting learning and preventing the formation of sufficiently differentiated activity patterns. These results indicate that effective pattern separation emerges from a balance between excitation-driven learning and inhibitory regulation.

**Fig. 5.**
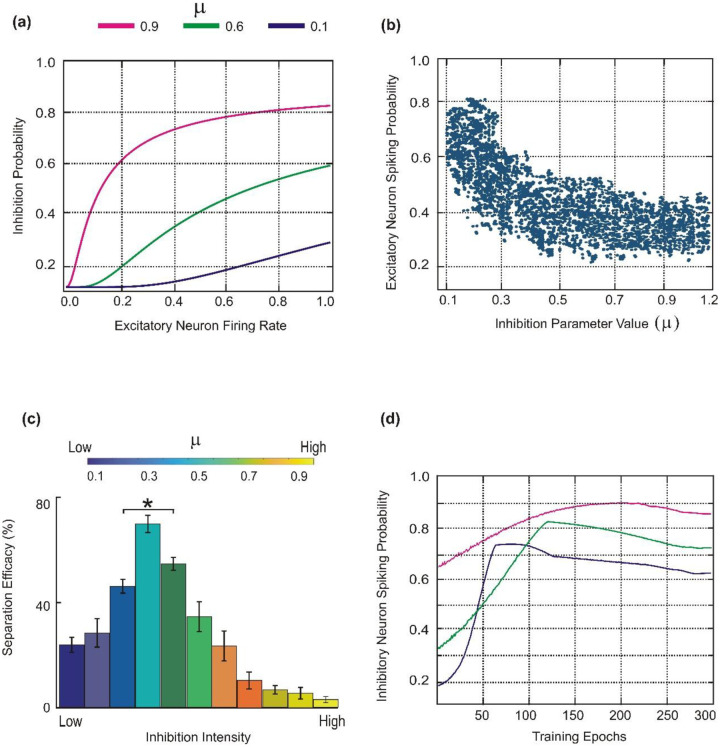
Effects of feedback inhibition on neural activity and pattern separation. **(a)** Feedback inhibition probability as a function of excitatory neuron firing rate for three inhibition parameter values (μ=0.1,0.6,and0.9). Increasing μ increases the probability of inhibitory firing across the range of excitatory activity, whereas lower μ values produce weaker inhibitory responses. **(b)** Relationship between inhibition intensity (μ) and excitatory neurons spiking probability. Excitatory activity decreases with increasing inhibition strength, revealing a significant negative correlation between inhibition intensity and excitatory spiking probability (Pearson’s r = −0.70, p < 0.001). **(c)** Pattern separation efficacy as a function of inhibition intensity (μ). Separation efficacy exhibits a non-monotonic dependence on inhibitory strength, peaking at moderate μ values, consistent with an optimal balance between excitation and inhibition. Bars represent mean separation efficacy across simulations, and error bars indicate SEM. The asterisk denotes a statistically significant difference between the indicated conditions (p < 0.05). Results were averaged over n = 50 independent simulations. **(d)** Evolution of the mean spiking probability of inhibitory neurons in the fourth layer during training for different inhibition intensities (μ=0.1,0.6,and0.9). Higher inhibition strengths produce higher sustained inhibitory activity, whereas lower inhibition strengths reach a plateau earlier and maintain lower inhibitory firing probabilities throughout training.

Feedback inhibition was modeled using a probabilistic inhibitory neuron whose firing probability was determined by Equation 7 (Fig. 5a). The inhibition parameter μ controlled inhibitory strength, with larger μ values producing stronger inhibitory responses. Excitatory neuron spiking probability decreased systematically as inhibition intensity increased (Fig. 5b), indicating a negative relationship between inhibitory strength and excitatory activity. Pattern separation efficacy exhibited a non-monotonic dependence on μ, reaching its maximum at moderate inhibition levels (Fig. 5c). The temporal evolution of inhibitory neuron activity also varied with μ (Fig. 5d), demonstrating how inhibition shapes network dynamics during learning and contributes to the emergence of optimal pattern separation performance.

**Fig. 6.**
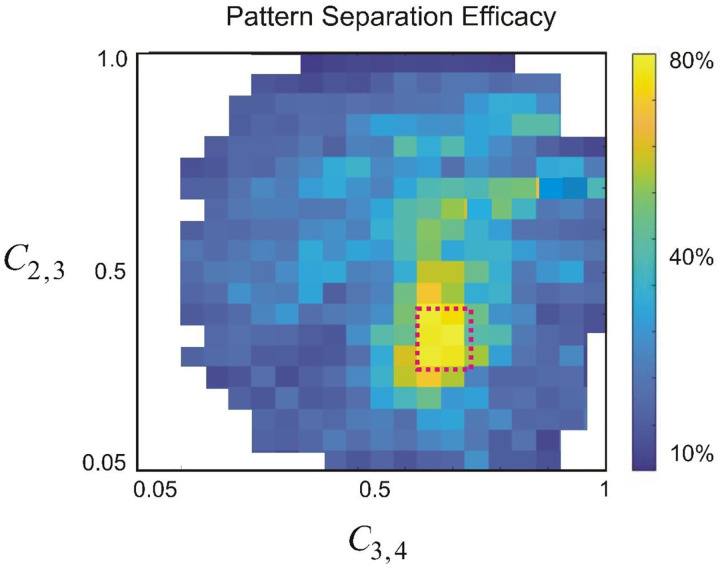
Maximum pattern separation efficacy across layer connectivity parameters. The heat map illustrates the maximum pattern separation efficacy achieved for different combinations of interlayer connectivity values $\left(C_{2\_3},C_{3\_4}\right)$. Warmer colors indicate higher efficacy, while cooler colors correspond to lower efficacy. The dashed red rectangle highlights the region associated with the highest pattern separation performance. Different levels of inhibitory regulation during training resulted in distinct connectivity configurations within this parameter space. The concentration of optimal performance within a relatively sparse connectivity regime supports the role of sparse coding in enhancing pattern separation in feedforward spiking neural networks.

Figure 6 summarizes the relationship between interlayer connectivity and pattern separation efficacy. The red-highlighted region denotes the range of connectivity values ${(C}_{2\_3},C_{3\_4})$ that produced the highest separation efficacy. Notably, optimal performance is concentrated within a relatively sparse yet functional connectivity regime, suggesting that excessive or insufficient connectivity can reduce pattern separation capability. These results reinforce the importance of sparse coding as a key organizational principle that promotes efficient information processing and robust pattern discrimination in feedforward spiking neural networks, consistent with observations in biological cortical circuits.

**Fig. 7.**
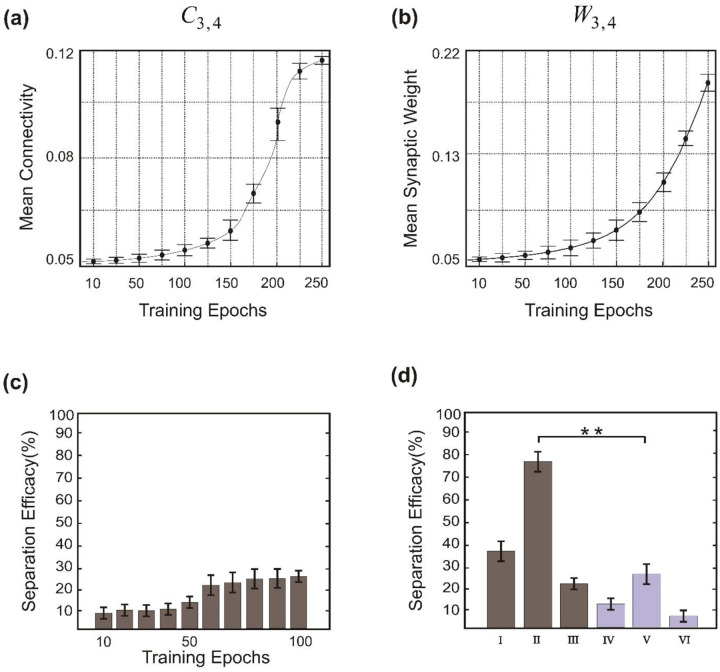
Comparison of synchronization-based and Hebbian learning rules for pattern separation efficacy. (**a**) Evolution of the connectivity parameter $\left(C_{3\_4}\right)$ across training epochs under the Hebbian learning rule. (**b**) Corresponding changes in the mean synaptic weight $\left(W_{3\_4}\right)$ during training. (**c**) Pattern separation efficacy obtained with the Hebbian learning rule as a function of training epoch. Panels (a–c) correspond to simulations performed under moderate inhibition (μ=0.6). (**d**) Maximum pattern separation efficacy obtained under different inhibition regimes and learning rules. Conditions correspond to: (I) low inhibition with synchronization-based learning, (II) moderate inhibition with synchronization-based learning, (III) high inhibition with synchronization-based learning, (IV) low inhibition with Hebbian learning, (V) moderate inhibition with Hebbian learning, and (VI) high inhibition with Hebbian learning. Moderate inhibition combined with synchronization-based learning produced the highest separation efficacy. Bars represent mean values across n=50 independent simulations, and error bars indicate SEM. Statistical significance was assessed using one-way ANOVA, with the indicated comparison showing a significant difference (p<0.01).

To evaluate the effectiveness of the proposed synchronization-based learning rule, we compared its performance with that of a conventional Hebbian plasticity mechanism. Under Hebbian learning, the growth of connectivity $\left(C_{3\_4}\right)$ and the corresponding synaptic weights $\left(W_{3\_4}\right)$ progressed gradually throughout training (Figs. 7a,b), resulting in relatively modest improvements in pattern separation efficacy (Fig. 7c). In contrast, the synchronization-based learning rule achieved substantially higher separation efficacy across all inhibition regimes (Fig. 7d). The best performance was observed under moderate inhibition, where synchronization-based learning significantly outperformed the corresponding Hebbian condition (p<0.01). These findings suggest that synchronization-dependent plasticity more effectively organizes network connectivity to support robust pattern separation than conventional Hebbian learning alone.

**Fig. 8.**
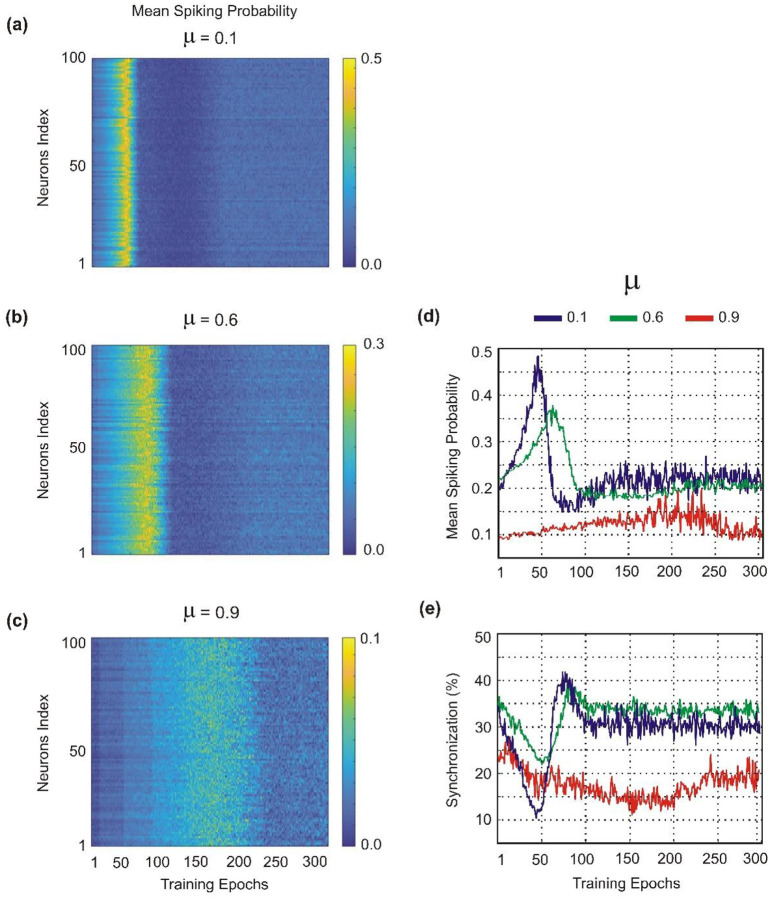
Firing activity and synchronization of output-layer excitatory neurons under different levels of feedback inhibition. (**a–c**) Heat maps showing the mean spiking probability of fourth-layer excitatory neurons across training epochs under (a) low inhibition (μ=0.1), (**b**) moderate inhibition (μ=0.6), and (**c**) high inhibition (μ=0.9). Color intensity represents the probability of neuronal firing. (**d**) Population-averaged spiking probability of fourth-layer excitatory neurons as a function of training epoch for the three inhibition regimes. (**e**) Average synchronization among fourth-layer excitatory neurons during training under low, moderate, and high inhibition. Moderate inhibition produced a transient increase in firing activity accompanied by the highest sustained synchronization, whereas excessive inhibition reduced both firing probability and synchrony. These results indicate that a moderate level of inhibition promotes coordinated network activity conducive to effective pattern separation.

We further investigated the relationship between neuronal activity, synchronization, and pattern separation performance under different levels of feedback inhibition. The spiking activity of fourth-layer excitatory neurons is shown in Figs. 8a–c for low, moderate, and high inhibition, respectively, with the corresponding population-averaged spiking probabilities presented in Fig. 8d. Low inhibition produced an early burst of activity followed by a decline, whereas high inhibition strongly suppressed firing throughout training. In contrast, moderate inhibition maintained a balanced level of activity that persisted over a broader training interval. The synchronization dynamics of the excitatory population are shown in Fig. 8e. Moderate inhibition yielded the highest sustained synchronization, while excessive inhibition reduced coordinated activity and low inhibition produced less stable synchronization patterns. Notably, the highest pattern separation efficacy observed in the network coincided with this moderate inhibitory regime, suggesting that a balance between excitation and inhibition promotes synchronized neural activity that supports efficient pattern separation.

**Fig. 9.**
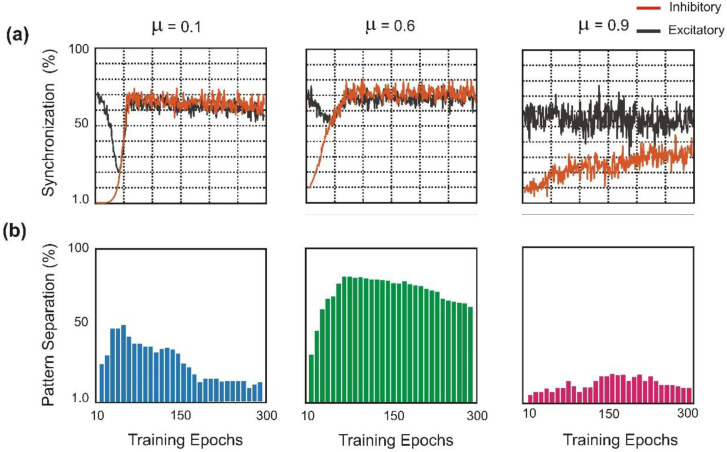
Relationship between excitatory–inhibitory synchronization and pattern separation efficacy. (**a**) Synchronization of fourth-layer excitatory (black) and inhibitory (red) neuronal populations during training under low (μ=0.1), moderate (μ=0.6), and high (μ=0.9) inhibition. Under low and moderate inhibition, excitatory and inhibitory populations progressively converge toward a coordinated synchronization regime, whereas strong inhibition maintains a persistent disparity between the two populations. (**b**) Pattern separation efficacy as a function of training epoch for the corresponding inhibition levels. The highest efficacy is observed under moderate inhibition, coinciding with sustained excitatory–inhibitory coordination. In contrast, low inhibition yields only transient improvements, while excessive inhibition results in poor separation performance throughout training. These results indicate that effective pattern separation is associated with a moderate regime of coordinated excitatory–inhibitory network activity.

To further investigate the relationship between network dynamics and computational performance, we analyzed the synchronization of excitatory and inhibitory populations together with pattern separation efficacy (Fig. 9). Under low (μ=0.1) and moderate (μ=0.6) inhibition, the synchronization levels of excitatory and inhibitory neurons gradually converged toward a coordinated regime during training, whereas strong inhibition (μ=0.9) prevented such convergence and maintained a pronounced separation between the two populations (Fig. 9a). The corresponding pattern separation efficacy is shown in Fig. 9b. Moderate inhibition produced the highest and most sustained separation efficacy, while low inhibition generated only a transient performance peak followed by a gradual decline. Under strong inhibition, pattern separation remained consistently low throughout training. Together, these findings indicate that optimal pattern separation emerges within a moderate regime characterized by coordinated excitatory–inhibitory activity, highlighting the importance of balanced network dynamics for efficient information processing.

**Fig. 10.**
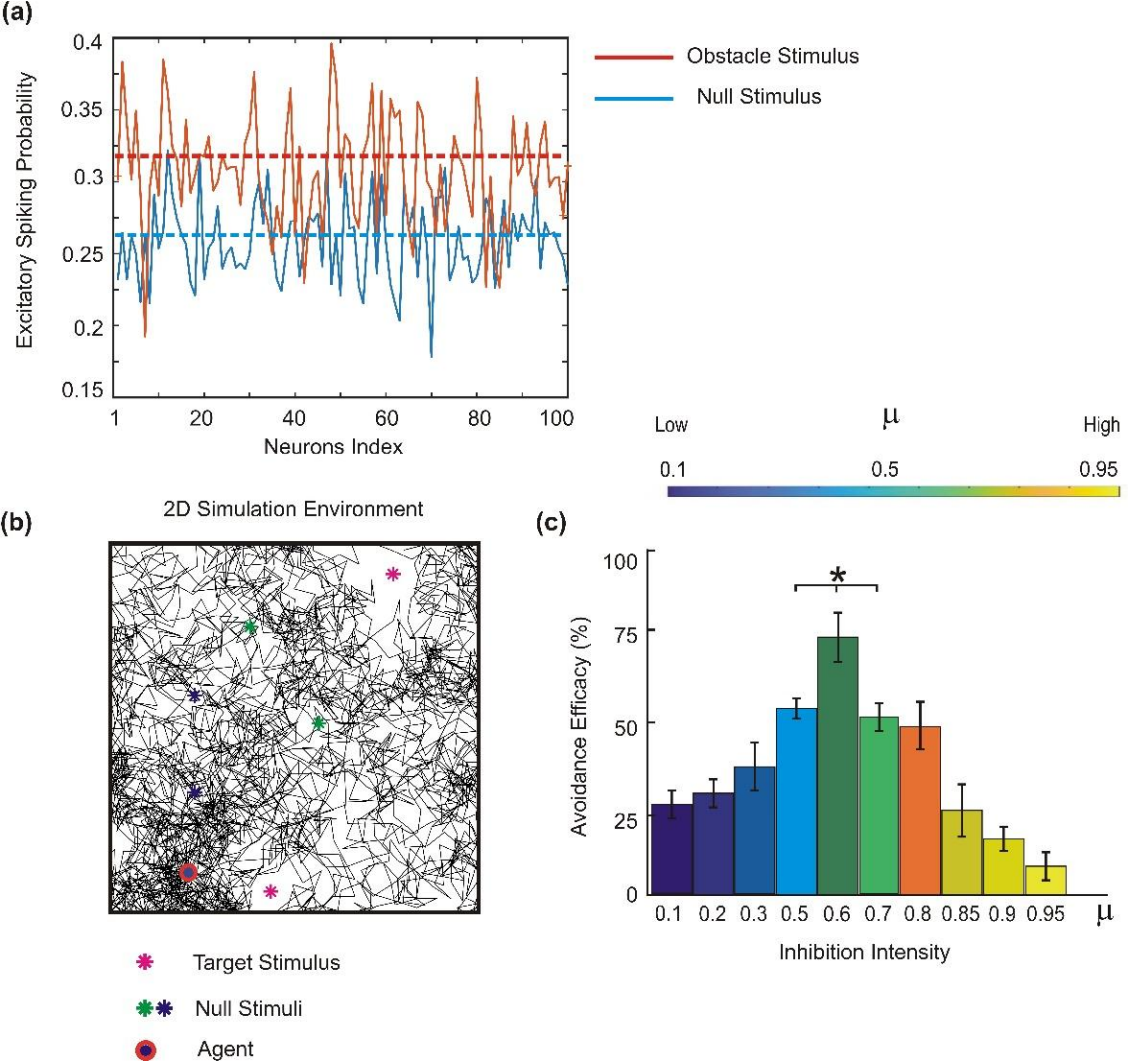
Obstacle detection and avoidance performance in a navigation task using the trained network. (a) Spiking probabilities of fourth-layer output neurons in response to obstacle and null stimuli. Horizontal dashed lines indicate the mean population response for each stimulus class. Obstacle stimuli evoke a stronger output-layer response than null stimuli, enabling discrimination based on population activity. (b) Representative trajectory of an artificial agent operating in a two-dimensional random-walk environment. The agent uses the trained network to classify encountered stimuli and modify its movement behavior accordingly. Magenta symbols denote target (obstacle) stimuli, green symbols denote null stimuli, and the red marker indicates the agent position. (c) Obstacle avoidance efficacy as a function of inhibition intensity (μ). Performance exhibits a non-monotonic dependence on inhibitory strength, reaching a maximum under moderate inhibition (μ=0.6). Bars represent mean avoidance efficacy across n = 50 independent simulations, and error bars indicate SEM. Statistical significance was assessed using one-way ANOVA followed by a post hoc multiple-comparison test; the indicated comparison was significant (p < 0.05).

To evaluate the functional capabilities of the trained network, we embedded it within an artificial navigation task. Analysis of the fourth-layer output neurons revealed distinct population responses to obstacle and non-obstacle (null) stimuli, both in terms of average firing probability and response variability across neurons (Fig. 10a). These differences enabled reliable stimulus classification using a threshold applied to the mean output-layer activity.

The trained network was then used to guide an artificial agent operating in a two-dimensional random-walk environment (Fig. 10b). During navigation, obstacle stimuli triggered avoidance behavior, whereas null stimuli allowed continued movement. Obstacle detection was therefore quantified by the agent’s ability to correctly identify and avoid obstacle stimuli encountered during exploration.

The effect of inhibitory strength on task performance is shown in Fig. 10c. Avoidance efficacy displayed a non-monotonic dependence on inhibition intensity, with the highest performance occurring under moderate inhibition (μ=0.6). Both weak and excessive inhibition reduced obstacle avoidance accuracy, consistent with previous analyses demonstrating that moderate inhibition optimizes pattern separation and stimulus discrimination. These results indicate that the connectivity and activity patterns emerging under moderate inhibition support not only enhanced pattern separation within the network but also improved behavioral performance in a dynamic navigation environment.

For each parameter setting, results were averaged across independent simulation runs, and the resulting performance measures were compared to identify the inhibition regime that maximized obstacle avoidance efficacy.

## Discussion

Our proposed synchronization-based learning rule is supported by previous studies demonstrating that neurons are highly sensitive to temporally synchronized synaptic input. In particular, earlier work^52^ showed that synchronized presynaptic activity can strongly modulate postsynaptic responses and influence information transmission within neural circuits. These findings support the central assumption of the present model that coincident spike timing carries biologically meaningful information capable of guiding synaptic reorganization and shaping large-scale network structure. Within this framework, synchronized input activity acts as a mechanism for selectively amplifying relevant signals during early stages of network development, prior to the emergence of mature activity-dependent plasticity mechanisms.

This study investigated the combined effects of feedback inhibition and a synchronization-based learning rule on self-organization and pattern separation within a probabilistic spiking neural network (PSNN). To our knowledge, synchronization-driven learning mechanisms remain relatively unexplored in artificial spiking neural networks. By systematically varying inhibitory strength, we identified a regime of moderate inhibition that optimized separation efficacy, network stability, and functional connectivity. These findings emphasize the importance of maintaining an appropriate balance between excitatory and inhibitory activity for efficient learning and information processing.

The results demonstrated that inhibitory strength plays a critical role in regulating network development. Excessive feedback inhibition suppressed a large fraction of the excitatory neuron population, limiting synaptic growth and impairing learning. In contrast, insufficient inhibition produced widespread neuronal activity, resulting in dense inter-layer connectivity and strong synaptic weights that reduced the network’s ability to discriminate between similar inputs. Moderate inhibition promoted a balanced interaction between activity and synaptic development, preventing both runaway excitation and excessive suppression. Networks operating within this regime developed sparse yet functionally effective connectivity patterns that supported robust stimulus discrimination. These observations are consistent with neurophysiological evidence suggesting that excitation–inhibition (E/I) balance is essential for maintaining stable neural activity while preserving representational diversity in cortical circuits.

Analysis of synchronization dynamics revealed that optimal performance coincided with the convergence of synchronization levels in excitatory and inhibitory populations. This coordinated state suggests that precise temporal interactions between neuronal populations facilitate efficient information encoding and transmission. Similar mechanisms have been observed in biological neural systems, where oscillatory synchronization contributes to memory formation, sensory processing, attention, and communication among distributed neural assemblies^53^. The present findings therefore support the view that synchronization serves not only as a marker of network organization but also as a functional mechanism contributing to adaptive learning.

Comparative analysis further demonstrated that the proposed synchronization-based learning rule outperformed conventional Hebbian learning. Networks trained using the Hebbian rule exhibited weaker connectivity development and reduced discrimination performance across inhibition conditions. By explicitly incorporating spike-timing relationships into synaptic adaptation, the proposed model captures an additional dimension of biologically plausible plasticity that is not represented in conventional rate-based learning mechanisms. These findings support the hypothesis that probabilistic and time-dependent plasticity rules contribute to the emergence of stable yet flexible network dynamics capable of supporting adaptive behavior^54^.

The observed relationship among inhibition, synchronization, and network performance highlights the importance of viewing learning in spiking neural systems as a dynamical process. Rather than relying solely on changes in synaptic strength, effective learning emerges from interactions among neuronal activity, connectivity, and temporal coordination. The results suggest that a dynamically balanced regime of excitation and inhibition allows the network to remain responsive to incoming stimuli while preserving overall stability. Such a regime provides a favorable substrate for the formation of sparse and informative internal representations, supporting both adaptability and robustness in neural computation.

Spiking neural networks provide a valuable framework for investigating neural mechanisms that are difficult to study directly in biological systems. Computational models enable systematic exploration of factors such as inter-layer connectivity, neuronal excitability, synchronization dynamics, and synaptic plasticity, offering insights into how these mechanisms contribute to learning and cognition. At the same time, SNNs provide a biologically inspired platform for testing hypotheses regarding neural development, self-organization, and information processing in large-scale networks.

Despite their biological realism and event-driven efficiency, SNNs continue to face important challenges in neuromorphic implementation. Local plasticity mechanisms such as spike-timing-dependent plasticity (STDP) often lack efficient optimization strategies comparable to gradient-based learning approaches, and performance in complex tasks^55^. The development of learning rules that preserve biological plausibility while enabling efficient self-organization therefore remains an important research objective.

Neuroscience research continues to seek a deeper understanding of how cognitive functions emerge through developmental processes. The mechanisms underlying self-organization, including synaptic plasticity and network optimization for efficient information processing, remain incompletely understood. Computational models such as the PSNN provide a useful framework for investigating these questions by enabling controlled manipulation of biological parameters and observation of their effects on network behavior. In this context, synchronization-based learning may represent one possible mechanism through which developing neural systems establish efficient functional connectivity while preserving stability and adaptability.

The ability to perform pattern separation has important implications beyond neuroscience. In artificial intelligence and robotics, distinguishing between highly similar inputs is essential for object recognition, environmental mapping, navigation, memory formation, and adaptive decision-making. Biological pattern separation mechanisms provide a useful source of inspiration for developing artificial systems capable of generalizing from previous experiences while minimizing interference among stored representations^56^.

These principles are particularly relevant to cognitive robotics, which seeks to develop autonomous systems capable of perception, learning, reasoning, and adaptation through biologically inspired computational architectures^57,58^. Self-organizing neural networks governed by local learning rules can form internal representations without extensive external supervision, allowing robotic systems to adapt continuously to changing environments. Such capabilities are especially important for real-time applications that operate under constraints of energy consumption, memory, and computational resources. Compared with conventional deep learning approaches, event-driven spiking models offer advantages in computational efficiency, online learning, and compatibility with neuromorphic hardware^59–63^.

Recent advances in neuromorphic computing further strengthen the relevance of these approaches. Event-driven hardware platforms are capable of processing information with substantially lower energy consumption than traditional computing architectures, making them attractive for mobile and autonomous cognitive systems. The integration of self-organizing spiking networks with neuromorphic hardware may therefore provide a pathway toward more efficient and adaptable intelligent agents capable of operating in dynamic real-world environments^63^.

Several limitations of the present study should be acknowledged. First, the model employs simplified probabilistic spiking neurons and therefore does not capture the full biophysical complexity of cortical neurons and synapses. Second, the network architecture is primarily feedforward and does not include recurrent connectivity, which is known to play an important role in memory formation, decision-making, and higher cognitive functions. Third, pattern separation was evaluated using synthetic input patterns rather than naturalistic sensory stimuli. Consequently, the generalizability of the proposed learning mechanism to more complex environments remains to be established. Future studies should examine whether synchronization-based learning can support similar computational advantages in recurrent architectures, larger-scale networks, and more realistic cognitive tasks.

A promising direction for future work is to reinterpret the proposed synchronization-based learning mechanism within predictive coding and free-energy minimization frameworks. Under this perspective, synchronized presynaptic activity may represent statistically reliable sensory evidence, while the synchronization term could act as a precision-weighting signal that regulates synaptic adaptation according to the consistency of incoming spike patterns. In addition, back-propagating spikes may serve as local error signals, conveying information about mismatches between expected and observed network activity, analogous to prediction-error signals in predictive coding models. Synaptic plasticity would therefore preferentially strengthen inputs contributing to coherent population dynamics while suppressing uncorrelated activity, thereby reducing representational uncertainty. The interaction between synchronized feedforward activity and error-related back-propagating spikes could provide a biologically plausible mechanism for local credit assignment and adaptive network reorganization. Such an interpretation may provide a biologically plausible bridge between sparse spiking neural networks, predictive coding, local error-driven learning, and free-energy minimization principles, potentially extending the applicability of the proposed model to cognitive neuroscience, neuromorphic computing, and brain-inspired artificial intelligence systems.

## Data Availability

The datasets generated and analyzed during the current study, along with the code used, are available from the corresponding author upon reasonable request.
